# Supplementary material for: MetaRibo-Seq measures translation in microbiomes
Source: Nat Commun. 2020 Jun 29;11:3268. doi: 10.1038/s41467-020-17081-z (PMC7324362; doi:10.1038/s41467-020-17081-z)
Supplement: Supplementary file 10 — Supplementary Data 7 [file 41467_2020_17081_MOESM10_ESM.zip › File2/Confidence_High_Taxonomy/58856_out.krona.html]

Javascript must be enabled to view this page.

members
magnitude
magnitudeUnassigned
count
unassigned
taxon
rank

58856\_out

4

2
4
superkingdom

1239
2
phylum
1

SRS1041129\_contig\_number\_889

91061
1
class

order

SRS058070\_contig\_number\_678
1
1385

2
544448
phylum

class
31969
2

order
2
2085

2
2092
family

2
2093
genus

1262905
2

SRS013476\_contig\_number\_8662SRS024132\_contig\_number\_1484
species
